# Supplementary material for: Prognostic Value of Systemic Immune-Inflammation Index (SII) in Patients with Glioblastoma: A Comprehensive Study Based on Meta-Analysis and Retrospective Single-Center Analysis
Source: J Clin Med. 2022 Dec 19;11(24):7514. doi: 10.3390/jcm11247514 (PMC9787672; doi:10.3390/jcm11247514)
Supplement: Supplementary file 1 [file jcm-11-07514-s001.zip › Supplementary Figures.pdf]

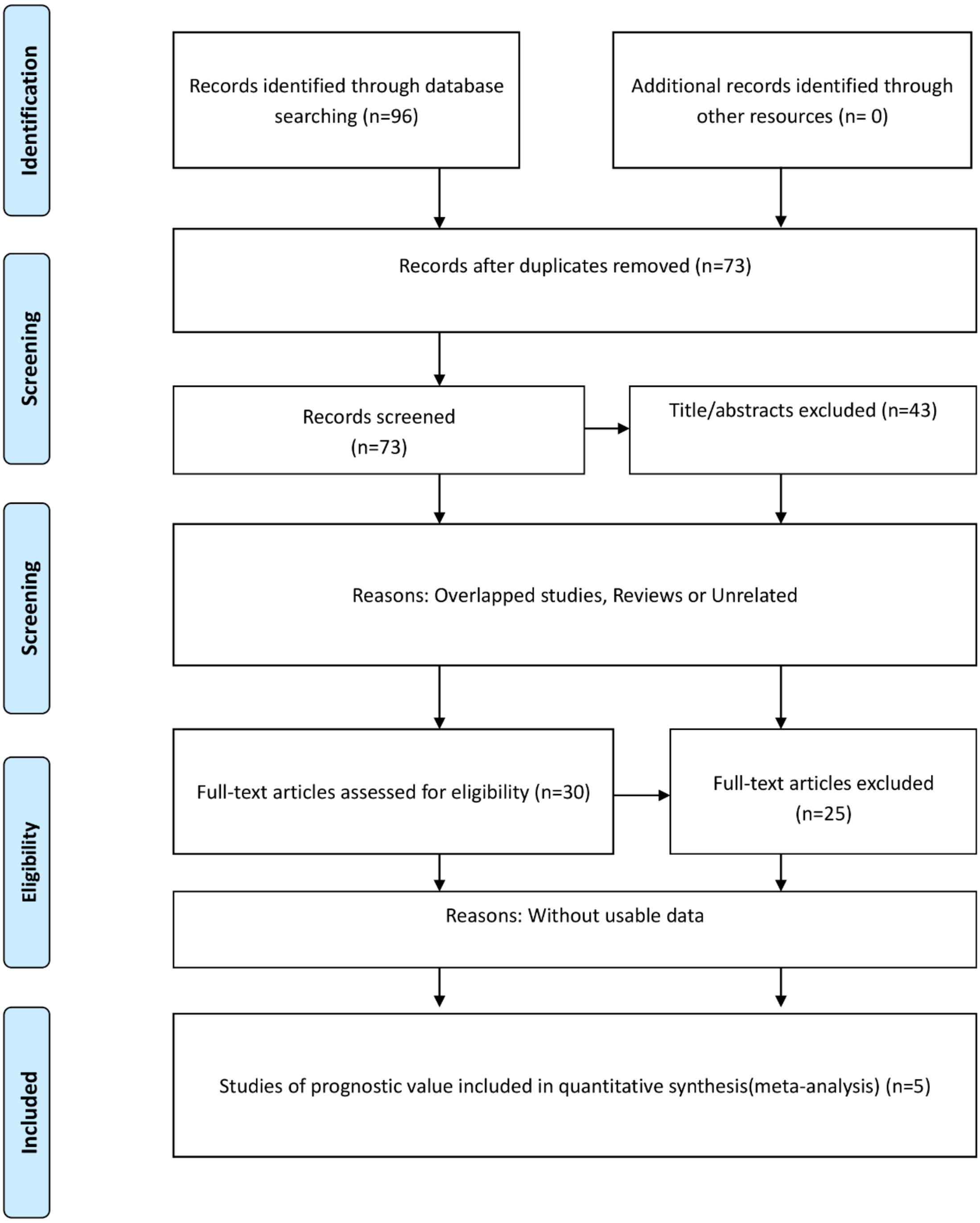

Supplementary Figure S1. Flow diagram of the study selection process during the meta-analysis

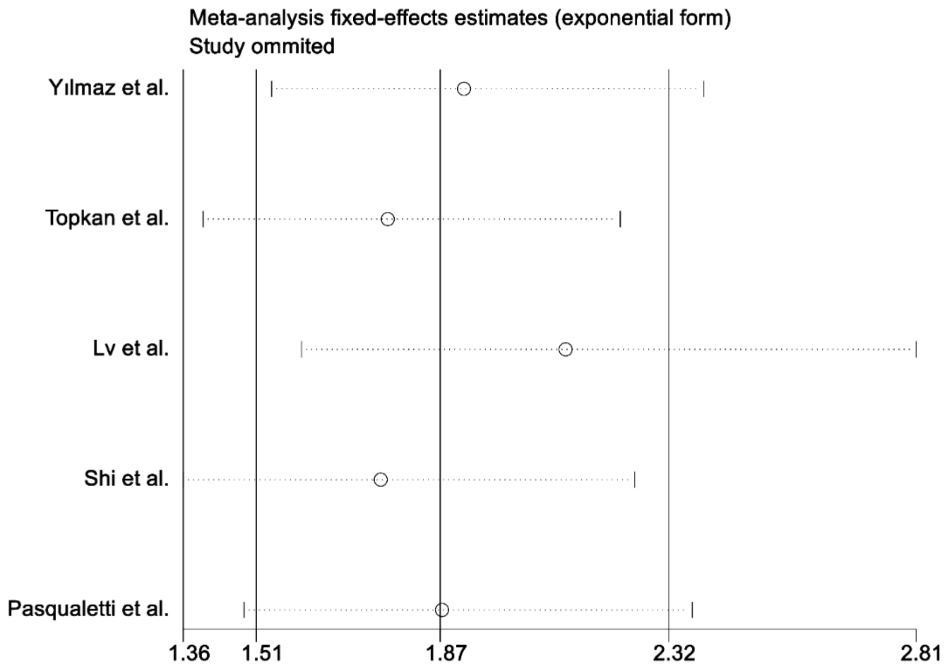

Supplementary Figure S2. Sensitive analysis of the relationship between SII and OS

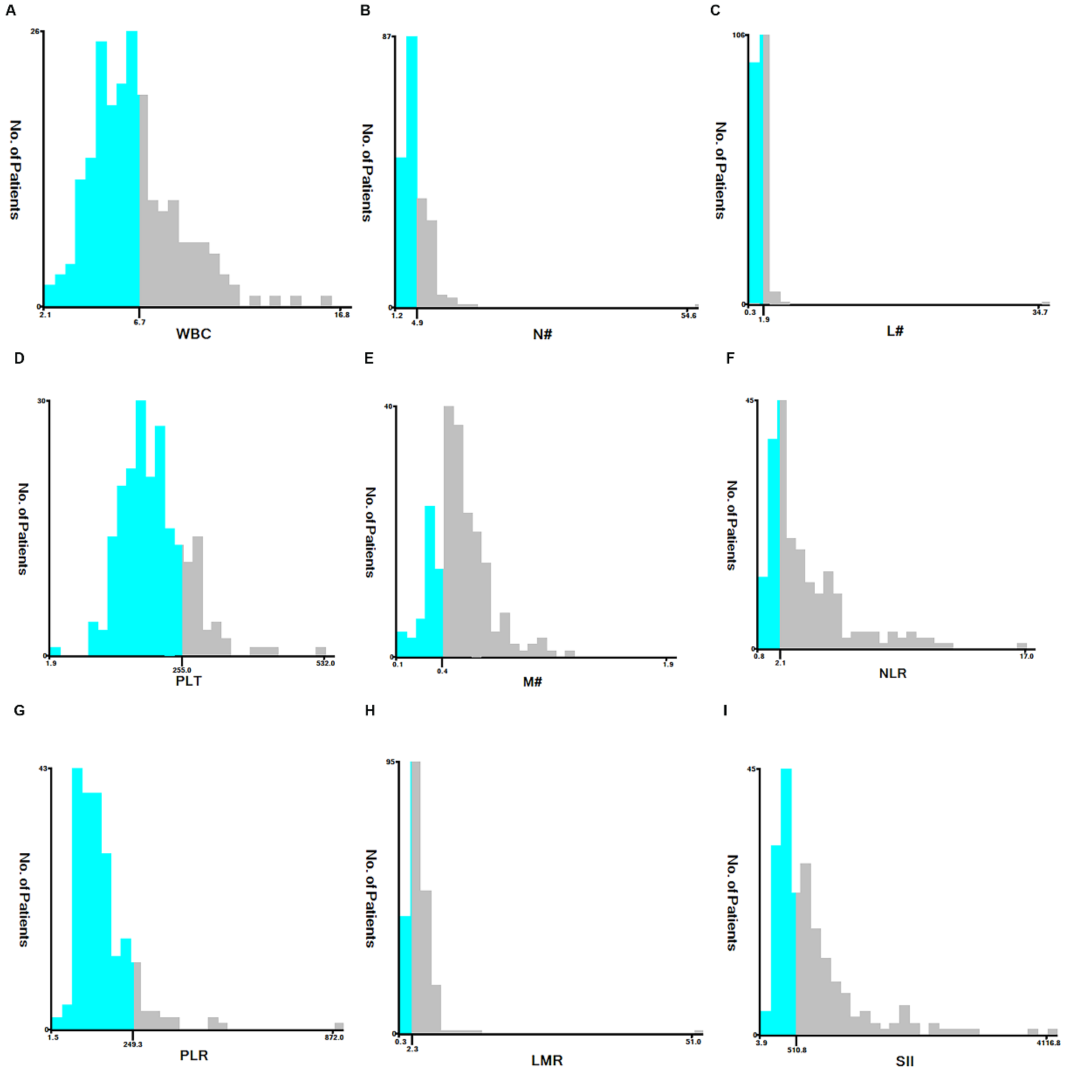

Supplementary Figure S3. Cut-off value of WBC **(A)**, neutrophil **(B)**, lymphocyte **(C)**, platelet **(D)**, monocyte **(E)**, NLR **(F)**, PLR **(G)**, LMR **(H)** and SII **(I)** in patients with GBM

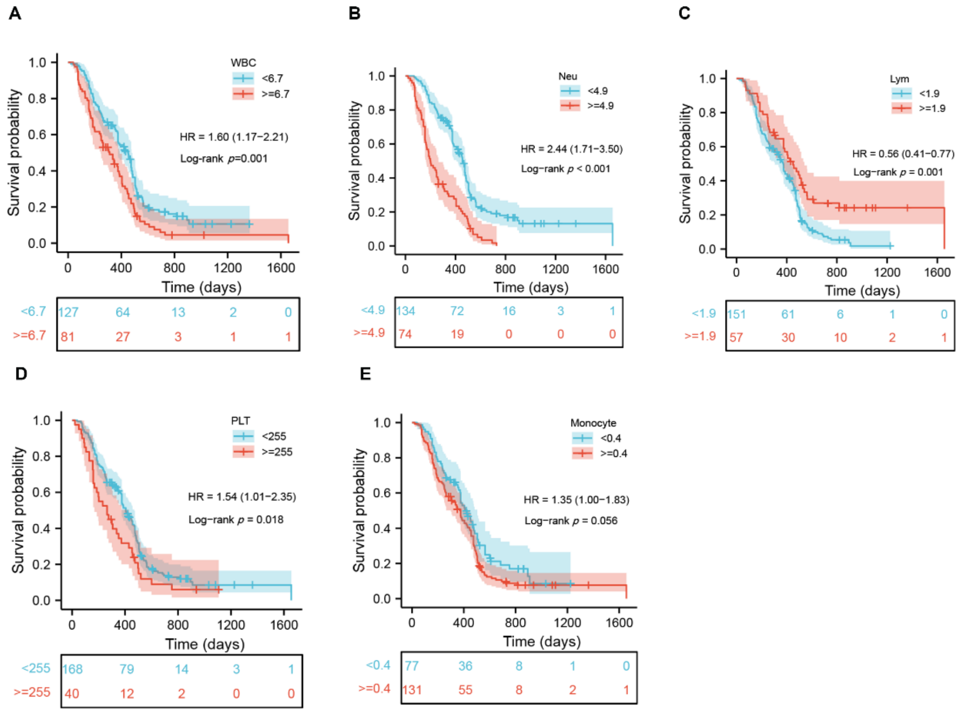

Supplementary Figure S4. Kaplan-Meier survival curves of glioblastoma patients based on the cutoff values of WBC **(A)**, neutrophil **(B)**, lymphocyte **(C)**, platelet **(D)** and monocyte **(E)**

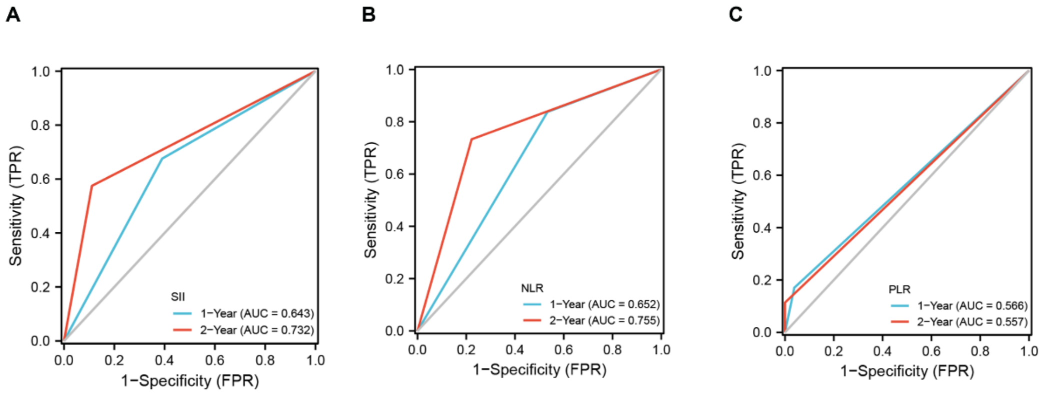

Supplementary Figure S5. Time-dependent ROC curve analysis of SII **(A)**, NLR **(B)** and PLR **(C)** for predicting OS at one-year and two-year survival rate in patients with GBM

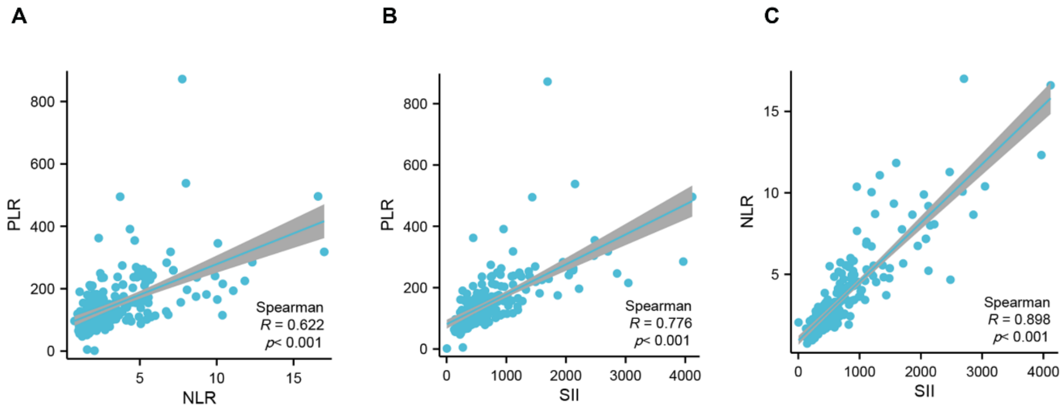

Supplementary Figure S6. Correlation analysis of SII, NLR and PLR.
