# Supplementary material for: Prognostic Value of Systemic Immune-Inflammation Index (SII) in Patients with Glioblastoma: A Comprehensive Study Based on Meta-Analysis and Retrospective Single-Center Analysis
Source: J Clin Med. 2022 Dec 19;11(24):7514. doi: 10.3390/jcm11247514 (PMC9787672; doi:10.3390/jcm11247514)
Supplement: Supplementary file 1 [file jcm-11-07514-s001.zip › Supplementary File S2.pdf]

Free terms: (1) “Glioblastomas”; “Astrocytoma, Grade IV”; “Astrocytomas, Grade IV”; “Grade IV Astrocytoma”; “Grade IV Astrocytomas”; “Glioblastoma Multiforme”; “Giant Cell Glioblastoma”; “Giant Cell Glioblastomas”; “Glioblastoma, Giant Cell”; “Glioblastomas, Giant Cell”.

(2) “SII”.
