# Supplementary material for: Prognostic Value of Systemic Immune-Inflammation Index (SII) in Patients with Glioblastoma: A Comprehensive Study Based on Meta-Analysis and Retrospective Single-Center Analysis
Source: J Clin Med. 2022 Dec 19;11(24):7514. doi: 10.3390/jcm11247514 (PMC9787672; doi:10.3390/jcm11247514)
Supplement: Supplementary file 1 [file jcm-11-07514-s001.zip › Supplementary File S4.pdf]

Statistical analysis for meta-analysis

The OS was analyzed by the HR. Forest plots were drawn to obtain the pooled HR, which was considered statistically significant if the 95% CI did not overlap 1 and the p value was less than 0.05. Subgroup analyzes were performed based on number of patients, sampling time and type of analysis to explore the sources of potential heterogeneity. Cochran's Q test and Higgins I-squared statistic were undertaken to assess the heterogeneity of the included studies and a P heterogeneity<0.10 or $I^2>50\%$ was considered as significant heterogeneity and random- or fixed-effect models were used when the heterogeneity was or was not significant. Sensitivity analysis was performed by excluding a single study at a time to examine the robustness of the results. Publication bias was evaluated by the Begg's and Egger's tests. All statistical processes of meta-analysis were performed using STATA statistical software (version 16.0; College Station, TX, USA).
