# Supplementary material for: Prognostic Value of Systemic Immune-Inflammation Index (SII) in Patients with Glioblastoma: A Comprehensive Study Based on Meta-Analysis and Retrospective Single-Center Analysis
Source: J Clin Med. 2022 Dec 19;11(24):7514. doi: 10.3390/jcm11247514 (PMC9787672; doi:10.3390/jcm11247514)
Supplement: Supplementary file 1 [file jcm-11-07514-s001.zip › Supplementary Tables.pdf]

Supplementary Table S1. Characteristics of the included prognostic studies

**Author (Year)**	**Country**	**N**	**Age**	**Grade**	**Sampling time**	**Cutoff value(10e 9/L)**	**UVA/MVA**
Lv (2019)	China	192	/	GBM	Preoperative	718	UVA
Topkan (2020)	Turkey	167	Median 57	GBM	Postoperative	565	MVA
Yılmaz (2021)	Turkey	120	/	GBM	Postoperative	1111	MVA
Shi(2022)	China	232	/	GBM	Preoperative	659.1	MVA
Pasqualetti(2022)	Italy	77	Median 64	GBM	Preoperative	1200	UVA

N(F/M): numbers of patients(female/male); HGG: high grade glioma; GBM: glioblastoma; UVA: univariate analysis; MVA: multivariate analysis

Supplementary Table S2. Quality evaluation of included studies using the Newcastle Ottawa Scale for cohort studies

Author	Year	Selections				Comparability	Outcome			Scores
		A	B	C	D	E	F	G	H	
Lv	2019	1	1	1	0	1	1	1	1	7
Topkan	2020	1	1	1	0	1	1	1	1	7
Yılmaz	2021	1	1	1	0	1	1	1	1	7
Shi	2022	1	1	1	0	1	1	1	1	7
Pasqualetti	2022	1	1	1	0	1	1	1	1	7

A: Representativeness of the exposed cohort: Highly representative or partially representative (one point); no description (no point), B: Selection of the non-exposed cohort: Patients drawn from the same community as the exposed cohort (one point); patients drawn from a different source or no description (no point), C: Ascertainment of exposure: Information obtained from secure record or structured interview (one point); written self-report or no description (no point),D: Demonstration that outcome of interest was not present at start of study: yes (one point), no (no point), E: Comparability of cohorts based on the design or analysis: Study controls for important factor, such as age, ECOG, molecular markers, extent of resection, (two points: All factors were included, one point: Part of them were included), F: Assessment of outcome: Independent blind assessment or record linkage (one point); self-report or no description (no point), G: Follow-up long enough for outcomes to occur: Yes (one point); no (no point), H: Adequacy of follow-up of cohorts: Complete follow up-all subjects accounted for or subjects lost to follow up unlikely to introduce bias (one point); no statement (no point).

Supplementary Table S3. Subgroup analysis of OS

Analysis		N	Ref.	Random-effects model		Fixed-effects model		Heterogeneity	
				**HR (95% CI)**	***p***	**HR(95% CI)**	***p***	**I^2(%)**	**Ph**
**Subgroup 1**	Preoperative	3	21,22,26			1.81(1.43-2.29)	<0.001	0	0.431
	Postoperative	2	20,23	1.92(0.79-4.67)	0.150	/	/	57.0	0.127
**Subgroup 2**	Sample size>100	4	20,21,23,26	/	/	1.87(1.48-2.36)	<0.001	32	0.220
	Sample size≤100	1	22	1.85(1.06-3.23)	0.020	/	/	/	/
**Subgroup 3**	MVA	3	20,21,23	/	/	2.21(1.60-3.07)	<0.001	14.3	0.311
	UVA	2	22,26	/	/	1.64(1.23-2.18)	0.001	0	0.624

N: number of studies; HR: hazard ratio; 95% CI: 95% confidence interval; Ph: p values of Q test for heterogeneity test; UVA: univariate analysis; MVA: multivariate analysis
